# Evolution and Molecular Control of Hybrid Incompatibility in Plants

**DOI:** 10.3389/fpls.2016.01208

**Published:** 2016-08-11

**Authors:** Chen Chen, Zhiguo E, Hong-Xuan Lin

**Affiliations:** ^1^Jiangsu Key Laboratory of Crop Genetics and Physiology, Co-Innovation Center for Modern Production Technology of Grain Crops, Key Laboratory of Plant Functional Genomics of the Ministry of Education, Yangzhou UniversityYangzhou, China; ^2^China National Rice Research InstituteHangzhou, China; ^3^National Key Laboratory of Plant Molecular Genetics and CAS Centre for Excellence in Molecular Plant Sciences, Shanghai Institute of Plant Physiology and Ecology, Shanghai Institutes for Biological Sciences, Chinese Academy of SciencesShanghai, China

**Keywords:** postzygotic reproductive barriers, Hybrid incompatibility, evolutionary force, Speciation genes, evolutionary genetics, Bateson-Dobzhansky–Muller model

## Abstract

Postzygotic reproductive isolation (RI) plays an important role in speciation. According to the stage at which it functions and the symptoms it displays, postzygotic RI can be called hybrid inviability, hybrid weakness or necrosis, hybrid sterility, or hybrid breakdown. In this review, we summarized new findings about hybrid incompatibilities in plants, most of which are from studies on *Arabidopsis* and rice. Recent progress suggests that hybrid incompatibility is a by-product of co-evolution either with “parasitic” selfish elements in the genome or with invasive microbes in the natural environment. We discuss the environmental influences on the expression of hybrid incompatibility and the possible effects of environment-dependent hybrid incompatibility on sympatric speciation. We also discuss the role of domestication on the evolution of hybrid incompatibilities.

## Reproductive Isolation and Postzygotic Reproductive Barriers

**FIGURE 1:**
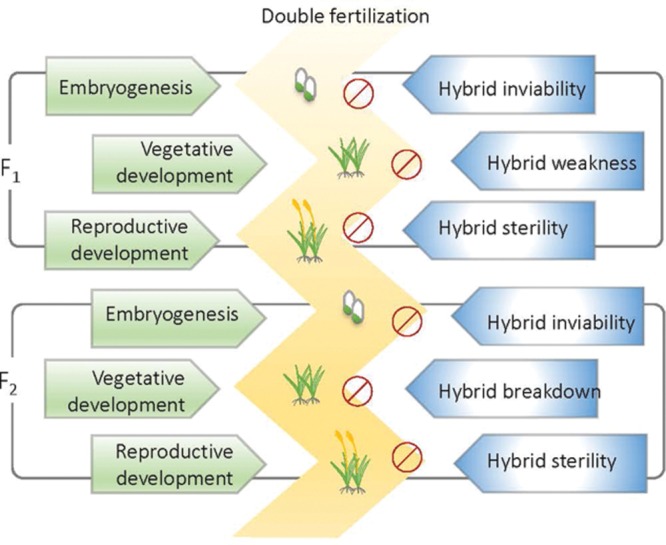
**Different forms of postzygotic reproductive isolation (RI)**.

Reproductive isolation (RI) hinders genetic exchange between species or populations. Therefore, it is considered fundamental to speciation (Coyne and Orr, 2004). Generally, RI in plants can be classified into prepollination and postpollination according to the developmental stage during which they occur. Prepollination isolations such as habitat divergence, temporal isolation, pollinator isolation, and mating system divergence usually function more effectively than postpollination isolations (Coyne and Orr, 2004). RI after postpollination can be divided into prezygotic isolation and postzygotic isolation. Postzygotic RI is common across the plant and animal kingdoms. Hybrids that undergo postzygotic RI are usually aborted or arrested after fertilization at different developmental stages or generations (**Figure 1**). According to the developmental stage at which postzygotic RI occurs and the symptoms it displays, postzygotic RI can be termed hybrid inviability, hybrid weakness or necrosis, hybrid sterility, or hybrid breakdown (**Figure 1**). In recent years, several genes involved in hybrid incompatibilities have been identified (Rieseberg and Blackman, 2010). These studies have extensively broadened our understanding of the molecular aspects of RI. In this review, we summarized new findings concerning the genetic and molecular control of intrinsic postzygotic hybrid incompatibility in plants and discuss the evolutionary basis of hybrid incompatibility in scenarios of evolution and domestication.

## The Bateson-Dobzhansky–Muller (BDM) Model and Genetic Incompatibility

**FIGURE 2:**
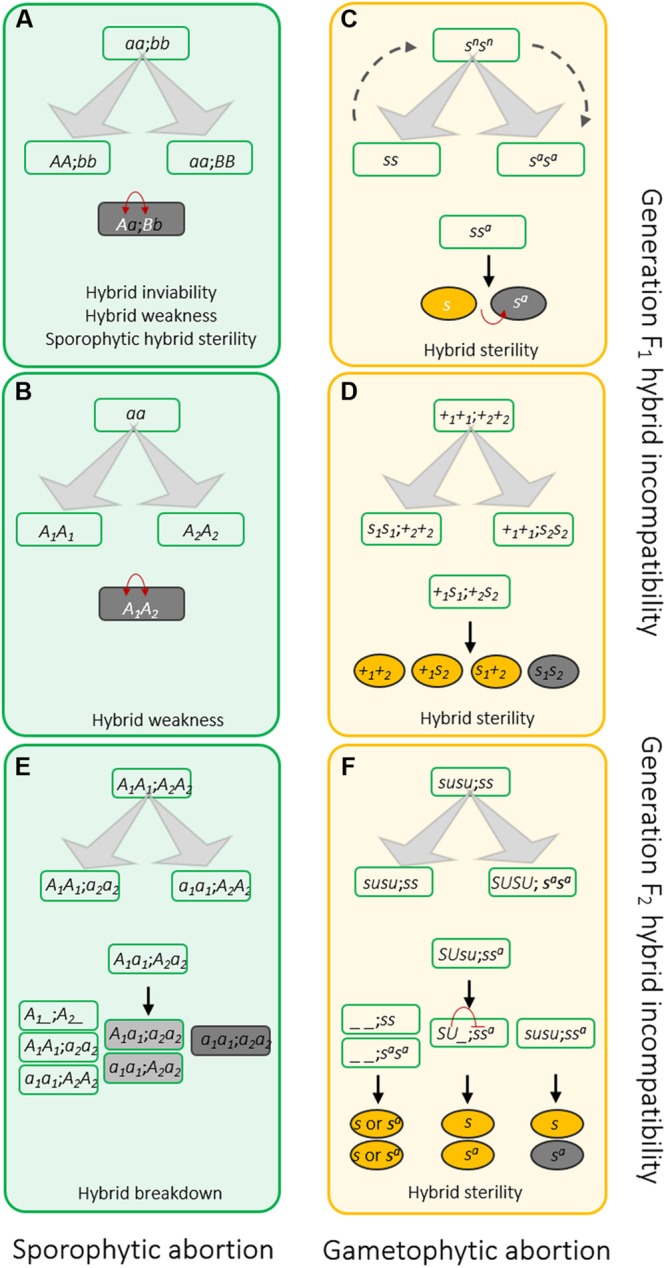
**Genetic models of hybrid incompatibilities that occur in sporophytes **(A,B,E)** and gametophytes **(C,D,F)** at *F*_1_ generation **(A–D)** or later generation **(E,F)**. (A)** Classic two-locus interaction Bateson-Dobzhansky–Muller (BDM) model. Ancestral genes were independently mutated and sustained in parallel populations isolated by geographic barrier. The mutations are compatible to their own genetic contexts. However, mixture of these mutations are deleterious to the hybrids and leads to hybrid incompatibility. **(B)** The allelic interaction BDM model. Distinct from the two-locus involved BDM model, disharmonious interaction occurs between different alleles at one locus. **(C)** The sporo-gametophytic interaction model. Allelic interaction cause gamete abortion in the hybrids. The selfish nature of *S* allele can kill gametes with genotype *S^a^*. The *S* and *S^a^* alleles are likely evolved from a wild-compatible allele *S^n^*, which is compatible with either *S* and *S^a^*. No fertility issue can be observed in *SS^n^* and *S^a^S^n^* hybrids. Alternatively, *S^n^* allele may also be the intermediate form of *S* and *S^a^* during evolution. For example, after the ancestral allele *S* mutating to *S^n^*, a second mutation of *S^n^* produces *S^a^*. **(D)** The duplicate gametic lethal model. At least one functional copy of the duplicated loci of *S1* and *S2* is required for gamete development. To avoid confusing of *s_1_* and *s_2_*, the functional alleles (*S_1_* and *S_2_*) are illustrated as +_*1*_ and *+_*2*_* in the figure. **(E)** The duplicate recessive lethal model to elucidate genetic control of sporophytic *F*_2_ hybrid dysfunction. Duplicated *A*_1_ and *A*_2_ are essential to plants. Mutation at one locus is tolerated in plants due to the redundancy. However, if two populations carry mutations at different loci, their offspring with genotype *a*_1_*a*_1_*a*_2_*a*_2_ will show inferiority, owing to lack of any functional copy of *A*_1_ or *A*_2_. **(F)** A more complicated genetic model controlling *F*_2_ hybrid sterility identified in rice. During evolution, a regulator *Su* was evolved to suppress the selfish nature of the *S* allele described in **(C)** in a sporophytic manner. Only the *ss^a^* hybrids without *SU* showed sterility. The red arrows in **(A–C)** indicate deleterious genetic interactions between loci or alleles. The yellow and gray ovals in **(D–F)** indicate fertile and sterile pollens, respectively.

Because of its adverse effects, how hybrid incompatibility could be sustained in different species was an enigma to Darwin and his contemporaries. Fisher (1930) realized that geographical isolation is important for the establishment of hybrid incompatibility. In the early 20th century, Bateson, Dobzhansky, and Muller independently proposed the BDM model (**Figure 2A**) to give a genetic explanation of hybrid incompatibility (Coyne, 1992; Orr, 1996). The BDM model proposes that distinct variations accumulate in divergent populations. Some variations are not compatible with variations fixed in other populations although the loci or genes involved are compatible with their native genetic context. The deleterious effects of the incompatible variations cause hybrid failure and prevent gene flow (**Figure 2**). Therefore, genetic incompatibility is essential to the formation of postzygotic RI.

By comprehensively reviewing the literature, Presgraves (2010) suggested that selfish genetic elements such as repeat sequences, transposable elements, and meiotic drivers are likely to be the main cause of hybrid incompatibility. This conclusion is applicable to plants as well if one broadly considers mitochondria and chloroplasts to be selfish elements. Mitochondria and chloroplasts evolved from ancient bacterial symbionts, which are dependent on host reproduction for their transmission and therefore, can be considered as “reproductive parasites” (Werren, 2011). Nucleocytoplasmic incompatibility can usually be found to cause hybrid sterility in either interspecies or intraspecies hybridization. However, unlike that found in *Drosophila* (Bayes and Malik, 2009; Ferree and Barbash, 2009; Phadnis and Orr, 2009), the vast majority of well characterized postzygotic RIs are not directly correlated with selfish genetic elements in plants (Rieseberg and Blackman, 2010; Ouyang and Zhang, 2013). This suggests that the genetic control of postzygotic RI in plants may be distinct from that in animals.

## Genetic and Molecular Regulation of Postzygotic RI

### Disruption of Genomic Imprinting Associates with Hybrid Inviability

Hybrid inviability, or hybrid lethality, is common in higher plants and is a particularly strong barrier to interspecific gene flow compared with other hybrid incompatibility forms (Coyne and Orr, 2004). Hybrid seed failure is usually caused by developmental defects in the endosperm (Lafon-Placette and Köhler, 2016). The endosperm and embryo are products of double fertilization. The triploid endosperm is needed to nourish the developing diploid embryo (Olsen, 2001). Histological observations suggest that endosperm breakdown is always coupled with endosperm cellularization defects (Walia et al., 2009; Ishikawa et al., 2011). Embryo rescue is an effective way to rescue the hybrids (Sharmal et al., 1996), indicating that the endosperm is less tolerant of genetic incompatibility than the embryo.

To date, our understanding of hybrid inviability is very limited in terms of its molecular controls. Hybrid inviability is not usually a reciprocal postzygotic hybridization barrier (Lafon-Placette and Köhler, 2016). As an example, unidirectional hybrid inviability in rice was found between W593A (*Oryza rufipogon*) and T65wx (*O. sativa* ssp. *japonica*) when W593A was the female parent and T65wx was the male parent (Matsubara et al., 2003). A genetic study revealed that the incompatibility is controlled by the maternal nuclear gene *Cif* and the paternal nuclear gene *cim* (Matsubara et al., 2003). The parent-of-origin dependent effects of incompatible nuclear genes indicate that epigenetic conflicts, most likely genomic imprinting, may lead to hybrid inviability. Imprinted genes are parent-of-origin dependent and regulate endosperm development in plants (Köhler et al., 2012). Imprinting interruption was discovered in the endosperm of interspecific and intraspecific animal and plant hybrids (Vrana et al., 1998; Josefsson et al., 2006; Ishikawa et al., 2011; Wolf et al., 2014; Burkart-Waco et al., 2015; Kirkbride et al., 2015). Misregulation of some imprinted genes, such as *MEDEA, FERTILIZATION*-*INDEPENDENT SEED 2*, and the type-I MADS box genes *PHERES1* and *AGAMOUS-LIKE 62*, has been associated with endosperm impairment in both interspecific and interploid *Arabidopsis* hybrids (Erilova et al., 2009; Walia et al., 2009; Jullien and Berger, 2010). Rice imprinted gene *OsMADS87* may play a similar role in the regulation of endosperm development (Ishikawa et al., 2011; Chen et al., 2016). Moreover, suppression of certain imprinted genes can alleviate the low viability of interspecific and interploid seeds (Walia et al., 2009; Kradolfer et al., 2013a,b; Wolff et al., 2015). The maintenance of genomic imprinting involves complex interactions between *trans*-acting factors and regulatory *cis*-elements (Bartolomei and Ferguson-Smith, 2011). Normal endosperm development requires a highly specific balance of gene expression (Gutierrez-Marcos et al., 2003). Mismatch of *trans-* and *cis-*components and/or misregulation of the imprinted-gene-regulated genes between species may result in disruption of the balance and impair endosperm development, which eventually causes hybrid lethality. Examples of hybrid inviability controlled by multiple loci have been found in several plants (Chu and Oka, 1970; Burkart-Waco et al., 2012; Garner et al., 2015; Rebernig et al., 2015). Cloning of these genes will broaden our understanding of the molecular control of hybrid inviability.

## Autoimmunity and Hybrid Weakness or Necrosis

Hybrid necrosis or weakness refers to the phenomena in which the hybrid shows developmental inferiority, such as necrotic leaves, small stature, or poor growth (Bomblies and Weigel, 2007). The weak hybrids often die before they reach the reproductive stage. Usually, the embryogenesis of plants expressing hybrid weakness is not affected (Saito et al., 2007; Chen et al., 2014). Classic genetic analysis indicated that deleterious BDM interactions between loci or alleles are the causes of hybrid necrosis and weakness (**Figures 2A,B**). In the past decade, several genes causing hybrid necrosis in plants have been cloned (Bomblies et al., 2007; Alcázar et al., 2009, 2010, 2014; Jeuken et al., 2009; Smith et al., 2011; Kradolfer et al., 2013a; Chen et al., 2014; Todesco et al., 2014), which comprehensively broaden our understandings of hybrid necrosis at molecular level. Almost all of the necrosis-causing genes identified from different plant species are likely to be associated with immune responses. In addition to the appearance of necrotic symptoms, pathogenesis-related genes are always activated (Bomblies et al., 2007; Alcázar et al., 2009; Chen et al., 2013). Though a few recessive genes have been found to induce the expression of hybrid necrosis (Bomblies and Weigel, 2007; Alcázar et al., 2009), most of the causal genes are dominant genes. This is consistent with the notion that most of the plant resistance genes are dominant genes. By systematically testing thousands of *F*_1_ hybrids, Chae et al. (2014) surprisingly found that there are autoimmune-caused hybrid necrosis hot spots in the *Arabidopsis* genome. Why the immune system is recruited in hybrid necrosis in various plant species is a fascinating but still open question. One reasonable explanation is that pathogens are a ubiquitous threat to plants. Microbe-driven selection accelerates the diversification of resistance genes (Jones and Dangl, 2006). The arm race between plants and pathogens facilitates the plant genome to accumulate more variations, making resistance genes one of the most diverse group of genes in plants (Bomblies, 2009). Due to fitness compensation, the immune system is delicately configured to ensure that defense responses will not be triggered unless invasion is detected. However, the diversity of defense-related genes substantially increases the risk of a defense regulation mismatch between different populations, which may activate defense responses in the absence of infection and cause hybrid necrosis and weakness as a fitness cost.

## Gene Duplication Plays Vital Role in Hybrid Dysfunction

The duplicate recessive lethal model can be used to elucidate the genetic control of sporophytic *F*_2_ hybrid inviability and breakdown (**Figure 2E**). After a duplication event, there are two copies of gene *A* (*A*_1_ and *A*_2_) in the ancestor genome. Due to redundancy, mutations that accumulate in either gene copy can be sustained in the genome. It is assumed that mutation of *A*_1_ is kept in population 1 (*a*_1_*a*_1_*A*_2_*A*_2_), and mutation of *A*_2_ is sustained in population 2 (*A*_1_*A*_1_*a*_2_*a*_2_). *A*_1_*a*_1_*A*_2_*a*_2_ hybrids and the majority of the *F*_2_ progeny will be viable, except for the ones with genotype *a*_1_*a*_1_*a*_2_*a*_2_, since they lack a functional copy. An example is the *HPA* gene, which encodes histidinol-phosphate aminotransferase involving histidine biosynthesis and therefore, is essential for plant growth and development (Bikard et al., 2009). The *Arabidopsis* genome has two *HPA* genes that were generated through a small chromosome segmental duplication. The *HPA* copy on chromosome 1 has been shown to be completely silenced in Columbia-0 (Col), while the other copy on chromosome 5 is deleted from the genome of Cape Verde Island accession Cvi-0 (Cvi). Therefore, approximately one-sixteenth of the seeds (*F*_2_) produced by Col/Cvi *F*_1_ hybrids, which lack a functional *HPA*, are unable to maintain essential development, which leads to embryo arrest (Bikard et al., 2009). Hybrid chlorosis, another type of hybrid incompatibility, has been found in several plant species (Tomar and Singh, 1998; Ichitani et al., 2012; Nakano et al., 2015). Linkage analysis of the *F*_2_ hybrid chlorosis genes in rice found that the genes are located on the distal regions of the short arms of chromosomes 12 and 11, respectively (Ichitani et al., 2012). Previous study showed that these regions are very conserved and were produced by chromosomal duplication (Wu et al., 1998). Therefore, this study may offer another case of disruption of duplicate genes causing hybrid incompatibility.

Oka (1988) conceived a similar model to explain the two loci-controlled *F*_1_ gametophytic sterility in rice. According to this model, two duplicated loci (*S*_1_ and *S*_2_) are important for gamete development. At least one dominant allele of these two loci (*+*_1_ or *+*_2_) is required. Therefore, 25% of the gametes produced by the hybrid (*S*_1_*s*_1_/*S*_2_*s*_2_), the ones with genotype *s*_1_*s*_2_, will be aborted due to a lack of *+*_1_ or *+*_2_. Recently, studies in rice provided molecular support for this model (Mizuta et al., 2010; Yamagata et al., 2010), which emphasizes a role of genetic drift in the evolution of hybrid incompatibility. As an example, a recent small-scale gene duplication event occurring after *Oryza–Brachypodium* differentiation was found to involve in the hybrid sterility of intraspecific rice hybrids (Mizuta et al., 2010). The disruption of duplicated gene *DOPPELGANGER1* (*DPL1*) in *japonica* rice cultivar Niponbare and *DPL2* in *indica* rice cultivar Kasalash causes pollen sterility in *F*_1_ hybrids (Mizuta et al., 2010). These findings confirm that gene duplication is important to hybrid incompatibility events and possibly to speciation.

## Complicated Genetic-Interaction-Networks Involve in Hybrid Sterility

Hybrid sterility is the most prevalent form of postzygotic RI in the plant and animal kingdoms (Ouyang et al., 2010; Rieseberg and Blackman, 2010; Maheshwari and Barbash, 2011). The hybrids develop vitally at the vegetative stage, whereas sterility appears in either the *F*_1_ or *F*_2_ generation. From a cytological perspective, fertility defects could be caused by degradation either of the generative cells (*n*), including pollen, the embryo-sac, or, in some cases, both, or of the vegetative/maternal tissues (2*n*) that enclose or surround the gametes, for instance, the tapetum cells. From this point of view, hybrid sterility could be sporophytic or gametophytic. Hybrid fertility determines the appearance of heterosis. Due to its practical importance, the genetic control of hybrid sterility has been comprehensively studied in crops. After six decades of study, more than 50 rice loci governing hybrid infertility have been identified to date (Kubo, 2013). Genetically, disharmonious allelic interaction is the major cause of *F*_1_ hybrid sterility, though sometimes, the deleterious interaction may involve two loci, which can be explained by Oka’s duplicate gametic lethal model aforementioned (**Figure 2D**).

The sporo-gametophytic interaction model (**Figure 2C**) proposed by Kitamura is a popular way to illustrate the allelic interactions that cause gametophytic sterility (Oka, 1988). The hybrid sterile gene (*S*) functions against its opposite allele (*S^a^*) in the hybrids (*S*/*S^a^*) during gametogenesis. Due to the selfish nature of *S*, the gametes carrying *S^a^* will be killed through an as-yet unidentified mechanism. Recently, a few of such hybrid sterility genes have been cloned and functionally characterized. The genes *S5* and *Sa* condition female and male sterility, respectively, in *indica*-*japonica* hybrids (Chen et al., 2008; Long et al., 2008; Yang et al., 2012). Initially, an aspartic protease-encoding gene that displays a two nucleotide difference between the *japonica* allele (*S5^j^*) and *indica* allele (*S5^i^*) was thought to be responsible for the incompatibility (Chen et al., 2008). Further study showed that two adjacent genes are also required to induce hybrid sterility (Yang et al., 2012). The three genes constitute a killer-protector system through switching on/off ER stress-induced premature programmed cell death in the embryo-sac, which determines megaspore fertility (Yang et al., 2012). Interestingly, the *Sa* locus also includes two adjacent genes, which encode a small ubiquitin-like modifier E3 ligase-like protein and an F-box protein, respectively (Long et al., 2008). *In vitro* experiments demonstrated that the products of the two causal genes can physically interact (Long et al., 2008). However, the underlying mechanism remains to be elucidated. These studies strongly suggest that this kind of pseudo-allelic interaction-controlled hybrid sterility may be more complicated than the sporo-gametophytic interaction model implies. In some cases, there exists a third allele, *S^n^*, that is compatible with either *S* or *S^a^* without a fertility issue in the hybrids (Oka, 1988). From an evolutionary perspective, *S^n^* may be the bridge between *S* and *S^a^* during evolutionary divergence. *S^n^* could either be the ancestral form of parallelly evolved *S* and *S^a^* or it may be the intermediate between sequentially evolved *S* and *S^a^*. Intriguingly, some gamete eliminators can be inactivated by an unlinked suppressor (Kubo et al., 2011). Such a suppressor, together with the gamete eliminator, could be employed as a neutral allele for evolution (**Figure 2F**).

*F*_2_ hybrid sterility is observed in various plant species (Kubo and Yoshimura, 2005; Sweigart et al., 2006; Yi et al., 2006; Li et al., 2015). Unlike *F*_1_ sterility, gamete abortion can be observed in some *F*_2_ individuals derived from a completely fertile *F*_1_ hybrid. Although the duplicate recessive lethal model (**Figure 2E**) can be applied to some sporophytic *F*_2_ hybrid sterility cases (Yi et al., 2010), recent studies in rice imply that the genetic control of *F*_2_ hybrid sterility can be more complicated (**Figure 2F**). As an example, *S24* was initially identified from chromosome segment substitution lines and acts gametophytically. In backcrossed *F*_1_, pollens carrying *japonica* allele *S24* (*S24^j^*) are killed due to the selfish nature of the *indica* allele (*S24^i^*) (Kubo et al., 2008). Nevertheless, further analysis revealed that the deleterious effect of *S24^i^* is also suppressed by another dominant sporophytic gene, *ESF*, from *indica* (Kubo et al., 2011). Therefore, *S24^i^* is unable to eliminate *S24^j^* in *indica*/*japonica F*_1_ hybrids because of the existence of *ESF*. However, in the segregated *F*_2_ population, *S24* is released from *ESF* in some progeny of the *esf* genotype, and this causes sterility at the *F*_2_ generation. Recently, another independent genetic pathway mediated by *S35* and *INK* was identified from the same parental derived population, indicating complicated genetic regulation of hybrid sterility (Kubo et al., 2016b). Interestingly, a recent study found that an initially reported *F*_2_ hybrid sterility causal locus *hsa1* of rice is composed by two tightly linked genes, and this locus may involve either *F*_1_ or *F*_2_ hybrid sterility events (Kubo et al., 2016a). This study identified a potential link between *F*_1_ and *F*_2_ sterility based on some common genetic elements. However, the combination of different elements in various germplasm may determine the genetic action manner of the genes.

Cytoplasmic male sterility (CMS) is another widely distributed type of hybrid sterility in many organisms. Plant CMS is determined by abnormal mitochondrial genes, which usually are gain-of-function open reading frames (ORFs) comprising segments derived from mitochondrial gene-coding and gene-flanking sequences and cause pollen failure of plants (reviewed in Chase, 2007). Many of these CMS mitochondrial genes can be suppressed or counteracted by the products of one or more nuclear encoded restorer genes. Several restorer genes have been identified in various plant species, most of which encoding pentatricopeptide-repeat proteins (PPRs; Bentolila et al., 2002; Brown et al., 2003; Desloire et al., 2003; Komori et al., 2004; Klein et al., 2005; Uyttewaal et al., 2008; Itabashi et al., 2011; Hu et al., 2012; Huang et al., 2015). These PPRs can suppress the accumulation of abnormal CMS mitochondrial transcripts (reviewed in Chase, 2007). Because mitochondria are usually maternally inherited, CMS is typically transmitted through female gametes, while restorer genes are transmitted through both male and female gametes. Mismatch or lack of restorer genes for the mitochondrial CMS gene in the hybrids will lead to hybrid sterility.

## Evolution of Hybrid Incompatibilities

An overview of the characterized speciation genes has shown that, in many cases, the evolution of hybrid incompatibility is likely not caused by adaptive mutations, but by arm races between selfish genetic elements and the host genes that regulate or suppress them (Presgraves, 2010). Notably, hybrid sterility is predominantly affected by selfish elements in animals (Presgraves, 2010; Maheshwari and Barbash, 2011). Selfish element-driven hybrid incompatibility is also prevalent in plant species and is responsible for the evolution of CMS (Rieseberg and Blackman, 2010). However, how selfish genetic elements can become fixed in a population remains to be elucidated because these elements, obviously, are usually detrimental to the host genome.

Several studies of plants have suggested that some genes responsible for hybrid necrosis and weakness are likely maintained by selection (Alcázar et al., 2010; Chen et al., 2014; Sicard et al., 2015). For instance, a rare allele of the *Arabidopsis Strubbelig Receptor Family 3* gene shows a recent selective sweep signature in Central Asian populations (Alcázar et al., 2010). Balancing selection was implied to act in the maintaining of the diversity of some hybrid necrosis causal genes in different species (Chen et al., 2014; Sicard et al., 2015). A recent study suggested that the *hms2* interacting BDM locus, *hms1*, of *Mimulus* causing hybrid sterility shows strong natural selection signature (Sweigart and Flagel, 2015). In addition, a gene responsible for incompatibility can be indirectly selected if the gene is physically adjacent to an adaptive gene (Presgraves, 2013). As an example, a population of yellow monkey flower has recently evolved an adaption to the tailings of local copper mines (Wright et al., 2013). When it crosses with off-mine plants, hybrid necrosis syndromes show an association with the copper tolerance. Recently, it was found that the necrosis locus *Nec1* is genetically linked with the tolerance locus *Tol1* (Wright et al., 2013). Hence, the selection pressure imposed by *Tol1* would help sustain *Nec1* in the mine-adapted population. Likewise, crop domestication may also increase the likelihood of genes causing incompatibility to fix in the population through genetic hitchhiking. For example, rice gamete eliminator *S5* is linked with *OsC1*, a gene targeted during domestication to control the color of the leaf sheath and apiculus (Saitoh et al., 2004).

These findings suggest that hybrid incompatibility is a by-product of co-evolution in the arm races between host and internal “parasitic” selfish elements or invasive microbes in the external environment. Different species have evolved distinct strategies to prevent invasion. Mismatch of regulation machineries is the cause of hybrid incompatibility *per se*. However, once a barrier is established, is it possible that selection directly favors the hybrid incompatibilities? Hybrid lethality is definitely disadvantageous. Nevertheless, the plants can sometimes derive benefit from it. If the hybrids are vigorous at the vegetative stage but are sterile or show weakness in the next generation, they occupy space and resources to compete with their parents. Under this circumstance, reproductive barriers that occur at an earlier stage may be selected to avoid producing inferior hybrids at later stages or the next generation. This allows the effective use of limited resources. For example, outbreeding species *O. longistaminata* often inhabits sympatrically with *O. breviligulata* and *O. glaberrima* (Oka, 1988). Hybrids between *O. longistaminata* and *O. breviligulata* or *O. glaberrima* usually suffer from hybrid inviability (Chu and Oka, 1970). A few seeds can occasionally be produced, but they are always weak and semisterile (Oka, 1988). Therefore, the complementary incompatible genes inducing hybrid inviability can be regionally increased to prevent resource consumption by the weak and sterile *F*_1_ plants. This is a possible explanation for why hybrid inviability is prevalent in wild rice of African origin.

## Environment and Hybrid Incompatibilities

Environmentally dependent genetic incompatibilities have been found in different species. By surveying the RI between 27 naturally compatible yeast isolates under 20 distinct environmental conditions, Hou et al. (2015) found that environment-dependent hybrid incompatibility is not rare. This conditionally expressed RI is also observed in plants. Most of the hybrid necrosis or weakness cases that have been studied so far are temperature sensitive (Bomblies et al., 2007; Saito et al., 2007; Alcázar et al., 2009; Fu et al., 2013; Chen et al., 2014). Usually, low temperature promotes the expression of inferior symptoms. This is consistent with the notion that high temperature can inhibit plant immunity (Traw and Bergelson, 2010; Alcázar and Parker, 2011; Hua, 2013). As an exceptional, contrasting case found in rice, *Hwi1*/*Hwi2*-induced hybrid weakness is suppressed by low temperature (Chen et al., 2014). For this case, the causal locus *Hwi1* consists of two indispensable *LRR-RLK* homologs, while its incompatible gene *Hwi2* encodes a secreted protease. This two-locus/three-gene system was assumed to overactivate defense responses through pattern-triggered immunity (PTI). A very interesting study recently showed that plants preferentially activate effector-triggered immunity (ETI) at low temperatures and PTI at high temperatures, possibly indicating that the genes involved in ETI are more likely to be recruited for establishing hybrid incompatibility between species (Cheng et al., 2013). Hybrid incompatibilities can also be affected by temperature in animals (Barbash et al., 2000; Koevoets et al., 2012). These examples suggest environmental factors such as temperature may interact with genetic control to determine the expression of hybrid incompatibilities.

**FIGURE 3:**
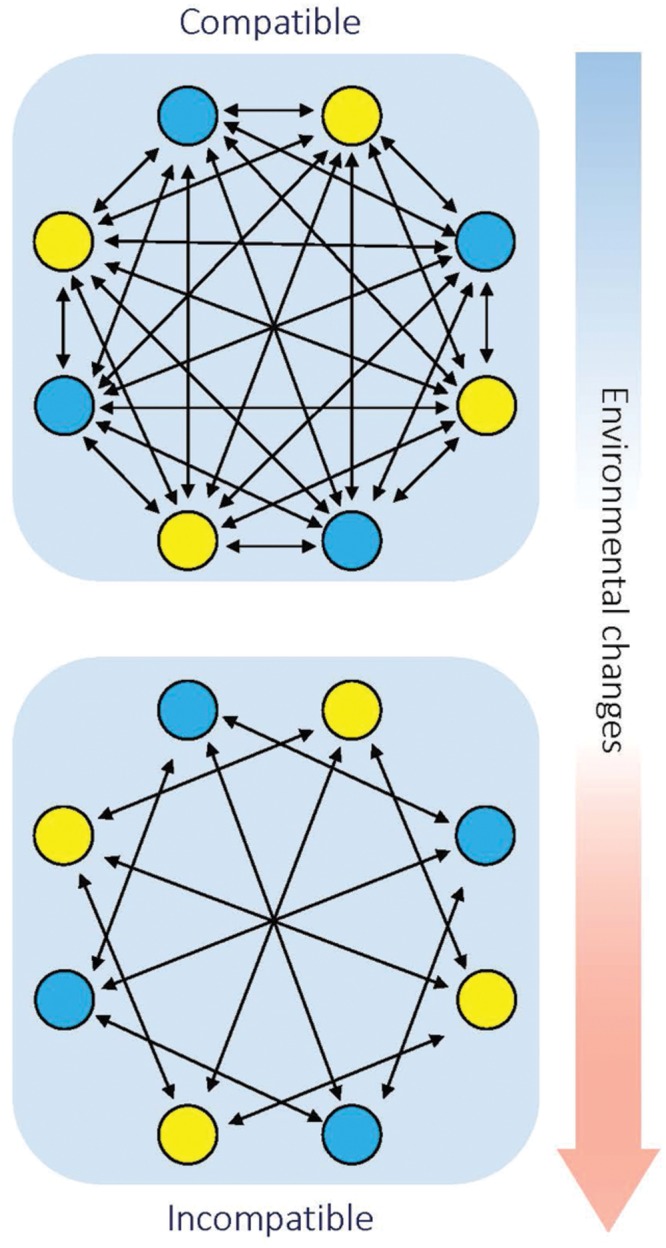
**Diagram of how environmentally conditioned hybrid incompatibility contributes to sympatric speciation.** Arrows indicate gene flow between individuals. Blue and yellow dots indicate individuals with different genotypes that inhabit the same region. Initially, individuals with different genotype are compatible with each other. Along with environmental changes (such as decreasing temperature), environmentally conditioned genetic incompatibility leads to hybrid incompatibility between different genotypes.

Geographical separation plays a vital role in speciation (Coyne and Orr, 2004). This is supported by the notion that most previously studied hybrid necrosis cases involve rare alleles found in geographically unrelated populations. However, at least one case found in *Arabidopsis* showed that the alleles involved co-exist in a certain place at high frequency, which means inferior hybrids can be observed in these populations in the wild (Todesco et al., 2014). As an explanation, the inferior hybrids may have a conditional advantage to maintain all the alleles in the population. However, once the adverse environment fails to persist, the alleles involved may contribute to sympatric speciation. Take hybrid necrosis as an example; populations inhabiting in the same niche carry different resistance genes that are maintained by selection. Initially, the local temperature is above the threshold to trigger the inferior symptoms such as necrosis. Thus, the populations are completely compatible. However, when the local climate changes and the temperature decreases, the previously compatible genes become incompatible and hinder genetic flow between the populations (**Figure 3**). Next, we need more evidences to support this hypothesis.

## Domestication and Crop Improvement May Contribute to Hybrid Incompatibility

Hybrid incompatibility is a by-product of evolution (Coyne and Orr, 2004). In terms of crops, artificial selection and hybridization accelerate the evolutionary process (Meyer and Purugganan, 2013). A literature survey indicated that majority of the most economically important crops were isolated from their progenitors with the existence or evolution of prezygotic and/or postzygotic reproductive barriers, even though geographic isolation was absent during domestication, at least in the initial stages of domestication for most species (Dempewolf et al., 2012). More important to early human farmers, RI can facilitate the maintenance of gene combinations during domestication. By taking these into account, Dempewolf et al. (2012) raised the question whether RI could be viewed as a long-overlooked “domestication trait,” though this hypothesis requires more supporting evidence.

To date, a few genes of hybrid incompatibility in crops have been isolated, however, the evolutionary study of these genes are very limited. Among them, *S5*, one of the key determinants of *japonica*-*indica* sterility, is about 400 kb apart from *OsC1*, which is responsible for anthocyanin pigmentation of rice. Loss-of-function *OsC1* alleles were selected during domestication (Saitoh et al., 2004). Molecular population analysis revealed that *japonica* and *indica* rice have independent origins of *OsC1* (Saitoh et al., 2004). Interestingly, *japonica* and *indica* S5 alleles were differentiated before domestication (Yang et al., 2012). It is unknown whether *japonica* and *indica* specific *OsC1* alleles were co-fixed with different *S5* alleles in rice cultivars during domestication. However, it is reasonable to assume that the selection of *OsC1* imposed an indirect force to sustain different *S5* alleles in different rice subspecies. Similar situation is observed at *Sa* locus, which located within a genomic region with strong selective sweep signature in *japonica* as well as *indica* rice (Huang et al., 2012). Yield is a key trait for crop domestication and improvement. *Gn1a* is important for rice yield formation (Ashikari et al., 2005). It is found that artificial selection of *Gn1a* plays an important role in improving rice yields across different ecological regions (Wang et al., 2015). Notably, *Gn1a* is linked with *S35* that determines pollen sterility of *japonica-indica* hybrids (Kubo, 2013). It will be interesting to interpret how the artificial selection of *Gn1a* functions on *S35* during domestication. Breeding can also affect the distribution of incompatible genes in crops. An unambiguous example comes from the geographical study of wheat hybrid necrosis inducing gene *Ne2*. Due to genetic linkage with the brown rust resistance gene, the frequency of *Ne2* in modern varieties has risen considerably in the past 100 years (Pukhalskiy et al., 2000). Moreover, at least one example of tomato showed that the gene involves in hybrid incompatibility may be directly selected by breeders for crop improvement. A *Lycopersicon pimpinellifolium* gene *Cf-2* confers resistance to the fungus *Cladosporium Fulvum* in an *Rcr3* dependent manner (Krüger et al., 2002). *Rcr3* was maintained by balancing selection and co-evolved with *Cf-2* in wild tomato species (Hörger et al., 2012). *Cf-2* has been bred into cultivated tomato (*L. esculentum*) for resistance improvement. However, *Cf-2* of wild tomato and *Rcr3* of cultivated tomato are able to interact with each other to induce hybrid necrosis syndrome in the hybrids (Krüger et al., 2002).

Collectively, genetic hitchhiking effect and direct selection of the hybrid incompatible genes can contribute to the RI in the history of crop domestication and improvement. However, we should note that all the hybrid incompatible genes are pre-existing in nature, which means domestication and breeding programs may comprehensively change the distribution and sustention of hybrid incompatible genes, rather than create new ones. Cloning and evolutionary analysis of the RI causal genes may provide novel insights to our understanding of how domestication affects RI and speciation. Rice, as a model organism for plant sciences, is one of the most suitable crops to test this hypothesis. Asian cultivated rice has two subspecies, *japonica* and *indica*, that are not fully isolated and can be regarded as the early stage of species divergence. African cultivated rice was domesticated independently of Asian rice. RI is very common between these species, including their progenitors (Chu and Oka, 1970; Oka, 1988). Several loci responsible for the interspecies or intraspecies hybrid incompatibilities have been mapped on different chromosomes. Cloning these hybrid incompatibility genes and studying their molecular and biochemical control in rice, as well as other plants, will substantially help us understand the evolution of RI.

## Author Contributions

All authors listed, have made substantial, direct and intellectual contribution to the work, and approved it for publication.

## Conflict of Interest Statement

The authors declare that the research was conducted in the absence of any commercial or financial relationships that could be construed as a potential conflict of interest.

## References

[B1] Alcázar R. García A. V. Kronholm I. de Meaux J. Koornneef M. Parker J. E. (2010). Natural variation at strubbelig receptor kinase 3 drives immune-triggered incompatibilities between *Arabidopsis thaliana* accessions. *Nat. Genet.* 42 1135–1139. 10.1038/ng.704 21037570

[B2] Alcázar R. García A. V. Parker J. E. Reymond M. (2009). Incremental steps toward incompatibility revealed by *Arabidopsis* epistatic interactions modulating salicylic acid pathway activation. *Proc. Natl. Acad. Sci. U.S.A.* 106 334–339. 10.1073/pnas.0811734106 19106299 PMC2629243

[B3] Alcázar R. Parker J. E. (2011). The impact of temperature on balancing immune responsiveness and growth in *Arabidopsis*. *Trends Plant Sci.* 16 666–675. 10.1016/j.tplants.2011.09.001 21963982

[B4] Alcázar R. von Reth M. Bautor J. Chae E. Weigel D. Koornneef M. (2014). Analysis of a plant complex resistance gene locus underlying immune-related hybrid incompatibility and its occurrence in nature. *PLoS Genet.* 10:e1004848 10.1371/journal.pgen.1004848 PMC4263378 25503786

[B5] Ashikari M. Sakakibara H. Lin S. Yamamoto T. Takashi T. Nishimura A. (2005). Cytokinin oxidase regulates rice grain production. *Science* 309 741–745. 10.1126/science.1113373 15976269

[B6] Barbash D. A. Roote J. Ashburner M. (2000). The *Drosophila melanogaster* hybrid male rescue gene causes inviability in male and female species hybrids. *Genetics* 154 1747–1771. 10747067 10.1093/genetics/154.4.1747 PMC1461041

[B7] Bartolomei M. S. Ferguson-Smith A. C. (2011). Mammalian genomic imprinting. *Cold Spring Harb. Perspect. Biol.* 3:a002592 10.1101/cshperspect.a002592 PMC3119911 21576252

[B8] Bayes J. J. Malik H. S. (2009). Altered heterochromatin binding by a hybrid sterility protein in *Drosophila* sibling species. *Science* 326 1538–1541. 10.1126/science.1181756 19933102 PMC2987944

[B9] Bentolila S. Alfonso A. A. Hanson M. R. (2002). A pentatricopeptide repeat-containing gene restores fertility to cytoplasmic male-sterile plants. *Proc. Natl. Acad. Sci. U.S.A.* 99 10887–10892. 10.1073/pnas.102301599 12136123 PMC125068

[B10] Bikard D. Patel D. Le Metté C. Giorgi V. Camilleri C. Bennett M. J. (2009). Divergent evolution of duplicate genes leads to genetic incompatibilities within *A. thaliana*. *Science* 323 623–626. 10.1126/science.1165917 19179528

[B11] Bomblies K. (2009). Too much of a good thing? Hybrid necrosis as a by-product of plant immune system diversification. *Botany* 87 1013–1022.

[B12] Bomblies K. Lempe J. Epple P. Warthmann N. Lanz C. Dangl J. L. (2007). Autoimmune response as a mechanism for a Dobzhansky-Muller-type incompatibility syndrome in plants. *PLoS Biol.* 5:e236 10.1371/journal.pbio.0050236 PMC1964774 17803357

[B13] Bomblies K. Weigel D. (2007). Hybrid necrosis: autoimmunity as a potential gene-flow barrier in plant species. *Nat. Rev. Genet.* 8 382–393. 10.1038/nrg2082 17404584

[B14] Brown G. G. Formanová N. Jin H. Wargachuk R. Dendy C. Patil P. (2003). The radish Rfo restorer gene of Ogura cytoplasmic male sterility encodes a protein with multiple pentatricopeptide repeats. *Plant J.* 35 262–272. 10.1046/j.1365-313X.2003.01799.x 12848830

[B15] Burkart-Waco D. Josefsson C. Dilkes B. Kozloff N. Torjek O. Meyer R. (2012). Hybrid incompatibility in *Arabidopsis* is determined by a multiple-locus genetic network. *Plant Physiol.* 158 801–812. 10.1104/pp.111.188706 22135429 PMC3271768

[B16] Burkart-Waco D. Ngo K. Lieberman M. Comai L. (2015). Perturbation of parentally biased gene expression during interspecific hybridization. *PLoS ONE* 10:e0117293 10.1371/journal.pone.0117293 PMC4342222 25719202

[B17] Chae E. Bomblies K. Kim S. T. Karelina D. Zaidem M. Ossowski S. (2014). Species-wide genetic incompatibility analysis identifies immune genes as hot spots of deleterious epistasis. *Cell* 156 1341–1351. 10.1016/j.cell.2014.10.049 25467443 PMC4269942

[B18] Chase C. D. (2007). Cytoplasmic male sterility: a window to the world of plant mitochondrial-nuclear interactions. *Trends Genet.* 23 81–90. 10.1016/j.tig.2006.12.004 17188396

[B19] Chen C. Begcy K. Liu K. Folsom J. J. Wang Z. Zhang C. (2016). Heat stress yields a unique MADS box transcription factor in determining seed size and thermal sensitivity. *Plant Physiol.* 171 606–622. 10.1104/pp.15.01992 26936896 PMC4854699

[B20] Chen C. Chen H. Lin Y. S. Shen J. B. Shan J. X. Qi P. (2014). A two-locus interaction causes interspecific hybrid weakness in rice. *Nat. Commun.* 5:3357 10.1038/ncomms4357 PMC3948059 24556665

[B21] Chen C. Chen H. Shan J. X. Zhu M. Z. Shi M. Gao J. P. (2013). Genetic and physiological analysis of a novel type of interspecific hybrid weakness in rice. *Mol. Plant* 6 716–728. 10.1093/mp/sss146 23220941

[B22] Chen J. Ding J. Ouyang Y. Du H. Yang J. Cheng K. (2008). A triallelic system of S5 is a major regulator of the reproductive barrier and compatibility of indica-japonica hybrids in rice. *Proc. Natl. Acad. Sci. U.S.A.* 105 11436–11441. 10.1073/pnas.0804761105 18678896 PMC2516230

[B23] Cheng C. Gao X. Feng B. Sheen J. Shan L. He P. (2013). Plant immune response to pathogens differs with changing temperatures. *Nat. Commun.* 4:2530 10.1038/ncomms3530 PMC3901997 24067909

[B24] Chu Y. Oka H. (1970). The genetic basis of crossing barriers between *Oryza perennis* subsp. *barthii* and its related taxa. *Evolution* 24 135–144. 10.1111/j.1558-5646.1970.tb01746.x 28563019

[B25] Coyne J. A. (1992). Genetics and speciation. *Nature* 355 511–515. 10.1038/355511a0 1741030

[B26] Coyne J. A. Orr H. A. (2004). *Speciation.* Sunderland, MA: Sinauer Associates.

[B27] Dempewolf H. Hodgins K. A. Rummell S. E. Ellstrand N. C. Rieseberg L. H. (2012). Reproductive isolation during domestication. *Plant Cell* 24 2710–2717. 10.1105/tpc.112.100115 22773750 PMC3426109

[B28] Desloire S. Gherbi H. Laloui W. Marhadour S. Clouet V. Cattolico L. (2003). Identification of the fertility restoration locus, Rfo, in radish, as a member of the pentatricopeptide-repeat protein family. *EMBO Rep.* 4 588–594. 12740605 10.1038/sj.embor.embor848 PMC1319198

[B29] Erilova A. Brownfield L. Exner V. Rosa M. Twell D. Scheid O. M. (2009). Imprinting of the Polycomb group gene MEDEA serves as a ploidy sensor in *Arabidopsis*. *PLoS Genet.* 5:e1000663 10.1371/journal.pgen.1000663 PMC2738949 19779546

[B30] Ferree P. M. Barbash D. A. (2009). Species-specific heterochromatin prevents mitotic chromosome segregation to cause hybrid lethality in *Drosophila*. *PLoS Biol.* 7:e1000234 10.1371/journal.pbio.1000234 PMC2760206 19859525

[B31] Fisher R. F. (1930). *The Genetical Theory of Natural Selection.* Oxford: Oxford University Press.

[B32] Fu C. Y. Wang F. Sun B. R. Liu W. G. Li J. H. Deng R. F. (2013). Genetic and cytological analysis of a novel type of low temperature-dependent intrasubspecific hybrid weakness in rice. *PLoS ONE* 8:e73886 10.1371/journal.pone.0073886 PMC3758327 24023693

[B33] Garner A. G. Kenney A. M. Fishman L. Sweigart A. L. (2015). Genetic loci with parent-of-origin effects cause hybrid seed lethality between *Mimulus* species. *New Phytol.* 211 319–331. 26924810 10.1111/nph.13897

[B34] Gutierrez-Marcos J. F. Pennington P. D. Costa L. M. Dickinson H. G. (2003). Imprinting in the endosperm: a possible role in preventing wide hybridization. *Philos. Trans. R. Soc. Lond. B Biol. Sci.* 358 1105–1111. 10.1098/rstb.2003.1292 12831476 PMC1693205

[B35] Hörger A. C. Ilyas M. Stephan W. Tellier A. van der Hoorn R. A. L. Rose L. E. (2012). Balancing selection at the tomato RCR3 guardee gene family maintains variation in strength of pathogen defense. *PLoS Genet.* 8:e1002813 10.1371/journal.pgen.1002813 PMC3400550 22829777

[B36] Hou J. Friedrich A. Gounot J.-S. Schacherer J. (2015). Comprehensive survey of condition-specific reproductive isolation reveals genetic incompatibility in yeast. *Nat. Commun.* 6:7214 10.1038/ncomms8214 PMC4445460 26008139

[B37] Hu J. Wang K. Huang W. Liu G. Gao Y. Wang J. (2012). The rice pentatricopeptide repeat protein RF5 restores fertility in Hong-Lian cytoplasmic male-sterile lines via a complex with the glycine-rich protein GRP162. *Plant Cell* 24 109–122. 10.1105/tpc.111.093211 22247252 PMC3289560

[B38] Hua J. (2013). Modulation of plant immunity by light, circadian rhythm, and temperature. *Curr. Opin. Plant Biol.* 16 406–413. 10.1016/j.pbi.2013.06.017 23856082

[B39] Huang W. Yu C. Hu J. Wang L. Dan Z. Zhou W. (2015). Pentatricopeptide-repeat family protein RF6 functions with hexokinase 6 to rescue rice cytoplasmic male sterility. *Proc. Natl. Acad. Sci. U.S.A.* 112 14984–14989. 10.1073/pnas.1511748112 26578814 PMC4672834

[B40] Huang X. Kurata N. Wei X. Wang Z. X. Wang A. Zhao Q. (2012). A map of rice genome variation reveals the origin of cultivated rice. *Nature* 490 497–501. 10.1038/nature11532 23034647 PMC7518720

[B41] Ichitani K. Takemoto Y. Iiyama K. Taura S. Sato M. (2012). Chromosomal location of HCA1 and HCA2 hybrid chlorosis genes in rice. *Int. J. Plant Genomics* 2012:6490811 10.1155/2012/649081 PMC3303693 22500165

[B42] Ishikawa R. Ohnishi T. Kinoshita Y. Eiguchi M. Kurata N. Kinoshita T. (2011). Rice interspecies hybrids show precocious or delayed developmental transitions in the endosperm without change to the rate of syncytial nuclear division. *Plant J.* 65 798–806. 10.1111/j.1365-313X.2010.04466.x 21251103

[B43] Itabashi E. Iwata N. Fujii S. Kazama T. Toriyama K. (2011). The fertility restorer gene, Rf2 for lead rice-type cytoplasmic male sterility of rice encodes a mitochondrial glycine-rich protein. *Plant J.* 65 359–367. 10.1111/j.1365-313X.2010.04427.x 21265890

[B44] Jeuken M. J. W. Zhang N. W. McHale L. K. Pelgrom K. den Boer E. Lindhout P. (2009). Rin4 causes hybrid necrosis and race-specific resistance in an interspecific lettuce hybrid. *Plant Cell* 21 3368–3378. 10.1105/tpc.109.070334 19855048 PMC2782268

[B45] Jones J. D. G. Dangl J. L. (2006). The plant immune system. *Nature* 444 323–329. 10.1038/nature05286 17108957

[B46] Josefsson C. Dilkes B. Comai L. (2006). Parent-dependent loss of gene silencing during interspecies hybridization. *Curr. Biol.* 16 1322–1328. 10.1016/j.cub.2006.05.045 16824920

[B47] Jullien P. E. Berger F. (2010). Parental genome dosage imbalance deregulates imprinting in *Arabidopsis*. *PLoS Genet.* 6:e1000885 10.1371/journal.pgen.1000885 PMC2841625 20333248

[B48] Kirkbride R. C. Yu H. H. Nah G. Zhang C. Shi X. Chen Z. J. (2015). An epigenetic role for disrupted paternal gene expression in postzygotic seed abortion in *Arabidopsis* interspecific hybrids. *Mol. Plant* 8 1766–1775. 10.1016/j.molp.2015.09.009 26409189

[B49] Klein R. R. Klein P. E. Mullet J. E. Minx P. Rooney W. L. Schertz K. F. (2005). Fertility restorer locus Rf1 of sorghum (*Sorghum bicolor* L.) encodes a pentatricopeptide repeat protein not present in the colinear region of rice chromosome 12. *Theor. Appl. Genet.* 111 994–1012. 10.1007/s00122-005-2011-y 16078015

[B50] Koevoets T. Van De Zande L. Beukeboom L. W. (2012). Temperature stress increases hybrid incompatibilities in the parasitic wasp genus *Nasonia*. *J. Evol. Biol.* 25 304–316. 10.1111/j.1420-9101.2011.02424.x 22122234

[B51] Köhler C. Wolff P. Spillane C. (2012). Epigenetic mechanisms underlying genomic imprinting in plants. *Annu. Rev. Plant Biol.* 63 331–352. 10.1146/annurev-arplant-042811-105514 22404470

[B52] Komori T. Ohta S. Murai N. Takakura Y. Kuraya Y. Suzuki S. (2004). Map-based cloning of a fertility restorer gene, Rf-1 in rice (*Oryza sativa* L.). *Plant J.* 37 315–325. 14731253 10.1046/j.1365-313x.2003.01961.x

[B53] Kradolfer D. Hennig L. Köhler C. (2013a). Increased maternal genome dosage bypasses the requirement of the FIS polycomb repressive complex 2 in *Arabidopsis* seed development. *PLoS Genet.* 9:e1003163 10.1371/journal.pgen.1003163 PMC3542072 23326241

[B54] Kradolfer D. Wolff P. Jiang H. Siretskiy A. Kohler C. (2013b). An imprinted gene underlies postzygotic reproductive isolation in *Arabidopsis thaliana*. *Dev. Cell* 26 525–535. 10.1016/j.devcel.2013.08.006 24012484

[B55] Krüger J. Thomas C. M. Golstein C. Dixon M. S. Smoker M. Tang S. (2002). A tomato cysteine protease required for Cf-2-dependent disease resistance and suppression of autonecrosis. *Science* 296 744–747. 10.1126/science.1069288 11976458

[B56] Kubo T. (2013). Genetic mechanisms of postzygotic reproductive isolation: Aan epistatic network in rice. *Breed. Sci.* 63 359–366. 10.1270/jsbbs.63.359 24399907 PMC3859346

[B57] Kubo T. Takashi T. Ashikari M. Yoshimura A. Kurata N. (2016a). Two tightly linked genes at the hsa1 locus cause both F_1_ and F_2_ hybrid sterility in rice. *Mol. Plant* 9 221–232. 10.1016/j.molp.2015.09.014 26455463

[B58] Kubo T. Yamagata Y. Eguchi M. Yoshimura A. (2008). A novel epistatic interaction at two loci causing hybrid male sterility in an inter-subspecific cross of rice (*Oryza sativa* L.). *Genes Genet. Syst.* 83 443–453. 10.1266/ggs.83.443 19282622

[B59] Kubo T. Yoshimura A. (2005). Epistasis underlying female sterility detected in hybrid breakdown in a japonica-indica cross of rice (*Oryza sativa* L.). *Theor. Appl. Genet.* 110 346–355. 10.1007/s00122-004-1846-y 15549230

[B60] Kubo T. Yoshimura A. Kurata N. (2011). Hybrid male sterility in rice is due to epistatic interactions with a pollen killer locus. *Genetics* 189 1083–1092. 10.1534/genetics.111.132035 21868603 PMC3213363

[B61] Kubo T. Yoshimura A. Kurata N. (2016b). Pollen killer gene S35 function requires interaction with an activator that maps close to S24., another pollen killer gene in rice. *G3* 6 1459–1468. 10.1534/g3.116.027573 27172610 PMC4856096

[B62] Lafon-Placette C. Köhler C. (2016). Endosperm-based postzygotic hybridization barriers: developmental mechanisms and evolutionary drivers. *Mol. Ecol.* 25 2620–2629. 10.1111/mec.13552 26818717

[B63] Li R. Guo M. Lu Y. Yang Y. Liu M. Zhu Q. (2015). Genetic dissection of hybrid breakdown in an indica/japonica cross and fine mapping of a quantitative trait locus qSF-12 in rice (*Oryza sativa* L.). *Mol. Breed.* 35 1–12. 10.1007/s11032-015-0331-4

[B64] Long Y. Zhao L. Niu B. Su J. Wu H. Chen Y. (2008). Hybrid male sterility in rice controlled by interaction between divergent alleles of two adjacent genes. *Proc. Natl. Acad. Sci. U.S.A.* 105 18871–18876. 10.1073/pnas.0810108105 19033192 PMC2596266

[B65] Maheshwari S. Barbash D. A. (2011). The genetics of hybrid incompatibilities. *Annu. Rev. Genet.* 45 331–355. 10.1146/annurev-genet-110410-132514 21910629

[B66] Matsubara K. Khin-Thidar Sano Y. (2003). A gene block causing cross-incompatibility hidden in wild and cultivated rice. *Genetics* 165 343–352. 14504241 10.1093/genetics/165.1.343 PMC1462754

[B67] Meyer R. S. Purugganan M. D. (2013). Evolution of crop species: genetics of domestication and diversification. *Nat. Rev. Genet.* 14 840–852. 10.1038/nrg3605 24240513

[B68] Mizuta Y. Harushima Y. Kurata N. (2010). Rice pollen hybrid incompatibility caused by reciprocal gene loss of duplicated genes. *Proc. Natl. Acad. Sci. U.S.A.* 107 20417–20422. 10.1073/pnas.1003124107 21048083 PMC2996679

[B69] Nakano H. Mizuno N. Tosa Y. Yoshida K. Park P. Takumi S. (2015). Accelerated senescence and enhanced disease resistance in hybrid chlorosis lines derived from interspecific crosses between tetraploid wheat and *Aegilops tauschii*. *PLoS ONE* 10:e0121583 10.1371/journal.pone.0121583 PMC4373817 25806790

[B70] Oka H.-I. (ed.) (1988). *Origin of Cultivated Rice.* Amsterdam: Elsevier.

[B71] Olsen O.-A. (2001). ENDOSPERM DEVELOPMENT: Cellularization and cell fate specification. *Annu. Rev. Plant Physiol. Plant Mol. Biol.* 52 233–267. 10.1146/annurev.arplant.52.1.233 11337398

[B72] Orr H. A. (1996). Dobzhansky, Bateson, and the genetics of speciation. *Genetics* 144 1331–1335. 8978022 10.1093/genetics/144.4.1331 PMC1207686

[B73] Ouyang Y. Liu Y. G. Zhang Q. (2010). Hybrid sterility in plant: stories from rice. *Curr. Opin. Plant Biol.* 13 186–192. 10.1016/j.pbi.2010.01.002 20153244

[B74] Ouyang Y. Zhang Q. (2013). Understanding reproductive isolation based on the rice model. *Annu. Rev. Plant Biol.* 64 111–135. 10.1146/annurev-arplant-050312-120205 23638826

[B75] Phadnis N. Orr H. A. (2009). A single gene causes both male sterility and segregation distortion in *Drosophila* hybrids. *Science* 323 376–379. 10.1126/science.1163934 19074311 PMC2628965

[B76] Presgraves D. C. (2010). The molecular evolutionary basis of species formation. *Nat. Rev. Genet.* 11 175–180. 10.1038/nrg2718 20051985

[B77] Presgraves D. C. (2013). Hitchhiking to speciation. *PLoS Biol.* 11:e1001498 10.1371/journal.pbio.1001498 PMC3582502 23468596

[B78] Pukhalskiy V. A. Martynov S. P. Dobrotvorskaya T. V. (2000). Analysis of geographical and breeding-related distribution of hybrid necrosis genes in bread wheat (*Triticum aestivum* L.). *Euphytica* 114 233–240. 10.1023/A:1003915708448

[B79] Rebernig C. A. Lafon-Placette C. Hatorangan M. R. Slotte T. Köhler C. (2015). Non-reciprocal interspecies hybridization barriers in the *Capsella* genus are established in the Endosperm. *PLoS Genet.* 11:e1005295 10.1371/journal.pgen.1005295 PMC4472357 26086217

[B80] Rieseberg L. H. Blackman B. K. (2010). Speciation genes in plants. *Ann. Bot.* 106 439–455. 10.1093/aob/mcq126 20576737 PMC2924826

[B81] Saito T. Ichitani K. Suzuki T. Marubashi W. Kuboyama T. (2007). Developmental observation and high temperature rescue from hybrid weakness in a cross between Japanese rice cultivars and Peruvian rice cultivar “Jamaica.”. *Breed. Sci.* 57 281–288. 10.1270/jsbbs.57.281

[B82] Saitoh K. Onishi K. Mikami I. Thidar K. Sano Y. (2004). Allelic diversification at the C (OsC1) locus of wild and cultivated rice: Nnucleotide changes associated with phenotypes. *Genetics* 168 997–1007. 10.1534/genetics.103.018390 15514070 PMC1448844

[B83] Sharmal D. R. Kaur R. Kumar K. (1996). Embryo rescue in plants – a review. *Euphytica* 89 325–337.

[B84] Sicard A. Kappel C. Josephs E. B. Lee Y. W. Marona C. Stinchcombe J. R. (2015). Divergent sorting of a balanced ancestral polymorphism underlies the establishment of gene-flow barriers in *Capsella*. *Nat. Commun.* 6:7960 10.1038/ncomms8960 PMC4539569 26268845

[B85] Smith L. M. Bomblies K. Weigel D. (2011). Complex evolutionary events at a tandem cluster of *Arabidopsis thaliana* genes resulting in a single-locus genetic incompatibility. *PLoS Genet.* 7:e1002164 10.1371/journal.pgen.1002164 PMC3136440 21779175

[B86] Sweigart A. L. Fishman L. Willis J. H. (2006). A simple genetic incompatibility causes hybrid male sterility in mimulus. *Genetics* 172 2465–2479. 10.1534/genetics.105.053686 16415357 PMC1456371

[B87] Sweigart A. L. Flagel L. E. (2015). Evidence of natural selection acting on a polymorphic hybrid incompatibility locus in mimulus. *Genetics* 199 543–554. 10.1534/genetics.114.171819 25428983 PMC4317661

[B88] Todesco M. Kim S. T. Chae E. Bomblies K. Zaidem M. Smith L. M. (2014). Activation of the *Arabidopsis thaliana* immune system by combinations of common ACD6 Alleles. *PLoS Genet.* 10:e1004459 10.1371/journal.pgen.1004459 PMC4091793 25010663

[B89] Tomar S. M. S. Singh B. (1998). Hybrid chlorosis in wheat rye crosses. *Euphytica* 99 1–4. 10.1023/A:1018353816039

[B90] Traw M. B. Bergelson J. (2010). Plant immune system incompatibility and the distribution of enemies in natural hybrid zones. *Curr. Opin. Plant Biol.* 13 466–471. 10.1016/j.pbi.2010.04.009 20494612

[B91] Uyttewaal M. Arnal N. Quadrado M. Martin-Canadell A. Vrielynck N. Hiard S. (2008). Characterization of *Raphanus sativus* pentatricopeptide repeat proteins encoded by the fertility restorer locus for Ogura cytoplasmic male sterility. *Plant Cell* 20 3331–3345. 10.1105/tpc.107.057208 19098270 PMC2630448

[B92] Vrana P. B. Guan X. J. Ingram R. S. Tilghman S. M. (1998). Genomic imprinting is disrupted in interspecific Peromyscus hybrids. *Nat. Genet.* 20 362–365. 10.1038/3833 9843208

[B93] Walia H. Josefsson C. Dilkes B. Kirkbride R. Harada J. Comai L. (2009). Dosage-dependent deregulation of an AGAMOUS-LIKE gene cluster contributes to interspecific incompatibility. *Curr. Biol.* 19 1128–1132. 10.1016/j.cub.2009.05.068 19559614 PMC6754343

[B94] Wang J. Xu H. Li N. Fan F. Wang L. Zhu Y. (2015). Artificial selection of Gn1a plays an important role in improving rice yields across different ecological regions. *Rice* 8:37 10.1186/s12284-015-0071-4 PMC4681714 26677125

[B95] Werren J. H. (2011). Selfish genetic elements, genetic conflict, and evolutionary innovation. *Proc. Natl. Acad. Sci. U.S.A.* 108(Suppl.) 10863–10870. 10.1073/pnas.1102343108 21690392 PMC3131821

[B96] Wolf J. B. Oakey R. J. Feil R. (2014). Imprinted gene expression in hybrids: perturbed mechanisms and evolutionary implications. *Heredity (Edinb)* 113 167–175. 10.1038/hdy.2014.11 24619185 PMC4105451

[B97] Wolff P. Jiang H. Wang G. Santos-González J. Köhler C. (2015). Paternally expressed imprinted genes establish postzygotic hybridization barriers in *Arabidopsis thaliana*. *Elife* 4 1–14. 10.7554/eLife.10074 PMC4589659 26344545

[B98] Wright K. M. Lloyd D. Lowry D. B. Macnair M. R. Willis J. H. (2013). Indirect evolution of hybrid lethality due to linkage with selected locus in *Mimulus guttatus*. *PLoS Biol.* 11:e1001497 10.1371/journal.pbio.1001497 PMC3582499 23468595

[B99] Wu J. Kurata N. Tanoue H. Shimokawa T. Umehara Y. Yano M. (1998). Physical mapping of duplicated genomic regions of two chromosome ends in rice. *Genetics* 150 1595–1603. 9832535 10.1093/genetics/150.4.1595 PMC1460416

[B100] Yamagata Y. Yamamoto E. Aya K. Win K. T. Doi K. Sobrizal (2010). Mitochondrial gene in the nuclear genome induces reproductive barrier in rice. *Proc. Natl. Acad. Sci. U.S.A.* 107 1494–1499. 10.1073/pnas.0908283107 20080642 PMC2824375

[B101] Yang J. Zhao X. Cheng K. Du H. Ouyang Y. Chen J. (2012). A killer-protector system regulates both hybrid sterility and segregation distortion in rice. *Science* 337 1336–1340. 10.1126/science.1223702 22984070

[B102] Yi B. Chen Y. Lei S. Tu J. Fu T. (2006). Fine mapping of the recessive genic male-sterile gene (Bnms1) in *Brassica napus* L. *Theor. Appl. Genet.* 113 643–650. 10.1007/s00122-006-0328-9 16804725

[B103] Yi B. Zeng F. Lei S. Chen Y. Yao X. Zhu Y. (2010). Two duplicate CYP704B1-homologous genes BnMs1 and BnMs2 are required for pollen exine formation and tapetal development in *Brassica napus*. *Plant J.* 63 925–938. 10.1111/j.1365-313X.2010.04289.x 20598092

